# Myeloid Cells in Glioblastoma Microenvironment

**DOI:** 10.3390/cells10010018

**Published:** 2020-12-24

**Authors:** Alessandra De Leo, Alessio Ugolini, Filippo Veglia

**Affiliations:** 1 Department of Immuno-Oncology, H. Lee Moffitt Cancer Center and Research Institute, Tampa, FL 33612-9416, USA; alessandra.deleo@moffitt.org (A.D.L.); alessio.ugolini@moffitt.org (A.U.); 2 Department of Experimental Medicine, Sapienza University of Rome, 00185 Rome, Italy

**Keywords:** myeloid cells, tumor-associated macrophages, microglia, neutrophils, dendritic cells, myeloid-derived suppressor cells, glioma, brain cancers, glioblastoma

## Abstract

Glioblastoma (GBM) is the most aggressive, malignant primary brain tumor in adults. GBM is notoriously resistant to immunotherapy mainly due to its unique immune microenvironment. High dimensional data analysis reveals the extensive heterogeneity of immune components making up the GBM microenvironment. Myeloid cells are the most predominant contributors to the GBM microenvironment; these cells are critical regulators of immune and therapeutic responses to GBM. Here, we will review the most recent advances on the characteristics and functions of different populations of myeloid cells in GBM, including bone marrow-derived macrophages, microglia, myeloid-derived suppressor cells, dendritic cells, and neutrophils. Epigenetic, metabolic, and phenotypic peculiarities of microglia and bone marrow-derived macrophages will also be assessed. The final goal of this review will be to provide new insights into novel therapeutic approaches for specific targeting of myeloid cells to improve the efficacy of current treatments in GBM patients.

## 1. Introduction

Gliomas are the most common tumors of the central nervous system (CNS) that originate from transformed neural stem or progenitor glial cells. The World Health Organization (WHO) divided gliomas into groups based on histopathological characteristics: low-grade gliomas (LGG, grades I and II) are well differentiated, slow-growing tumors, whereas high-grade gliomas (HGG, grades III and IV) are less differentiated or anaplastic, and strongly infiltrate brain parenchyma [1,2]. Grade IV glioma or glioblastoma (GBM) is the most common and aggressive form of brain tumors in adults. GBM has an incidence of 18,000 cases in the United States [3]. Despite surgical resection, targeted radiotherapy and high-dose chemotherapy, patients still have a median overall survival of <15 months and a 5-year survival rate of less than 3% [4]. GBM is notoriously resistant to immunotherapy and no survival benefit has been observed in recurrent GBM patients [5,6]. Intrinsic and extrinsic mechanisms remarkably contribute to the failure of immunotherapies in patients with GBM [7,8].

The brain has long been recognized as an immune privileged tissue because of the restrictions imposed by the brain–blood barrier (BBB) [9]. However, this concept of immune privilege has been partially redefined and now the brain is proposed to be an immunologically distinct rather than an immune privileged organ [7]. It is now clear that there are functional lymphatic vessels in the CNS, and that varied types of leukocytes exist within the CNS.

Histopathological, flow, and mass cytometry, single cell RNA sequencing analyses in human and rodent experimental gliomas reveal an extensive heterogeneity of immune cells infiltrating glioma microenvironments [10,11,12,13]. The majority of immune cells in gliomas, including GBM, comprises a vast diversity of myeloid cells, which include bone marrow-derived macrophages (BMDMs), microglia, myeloid-derived suppressor cells (MDSCs), dendritic cells and neutrophils.

Depending on histopathological and transcriptomic features as well as somatic mutation in isocitrate dehydrogenase 1/2 (IDH1/2), the glioma microenvironment can display differences in its immune components. Lower and higher grade gliomas are considered “cold” tumors due to the limited infiltration of lymphocytes [14] and their low responses to different immunotherapy strategies [15]. Lymphopenia is suggested to be caused by the downregulation of Sphingosine-1-phosphate receptor 1 (S1PR1) expression in lymphocytes, which are retained in the bone marrow [16]. The gliomas present unique transcriptomic profiles, which allows discrimination of classic (CL), mesenchymal (MES), neural (N), and proneural (PN) tumors [2]. Whereas MES gliomas are associated with vascular disorders and with the accumulation of immune cells, PN tumors show a reduced immune infiltration and a better prognosis [17]. The methylation induced by IDH1/2 mutations represses the essential genes necessary for the recruitment and induction of the immune response. Patients with IDH mutation show lower immune infiltrates and better prognosis independently of grade, compared to IDH wild type (WT) gliomas [18].

Here, we will review the most recent advances on the features and functions of different populations of myeloid cells in GBM, including BMDMs, microglia, MDSCs, dendritic cells, and neutrophils. We will also analyze epigenetic, metabolic, and phenotypic peculiarities of microglia and BMDMs. The final goal of this review will be to provide new insights for designing alternative therapeutic approaches for specific targeting of myeloid cells in GBM patients to enhance the efficacy of current therapies for GBM patients.

## 2. Tumor-Associated Macrophages (TAMs)

Tumor-associated macrophages (TAMs) represent one of the most prominent populations in the tumor stroma and their abundance has been shown to correlate with clinical outcomes in many cancers [19]. TAMs in mouse and human cancers largely express molecules associated with M2-like phenotypes that include arginase (ARG1), interleukin-10 (IL-10), and transforming growth factor β (TGFβ), which induce immunosuppression and fibrosis within the tumor microenvironment [20]. Conversely, the prevalence of macrophages with M1-like phenotypes expressing IL-12, Tumor Necrosis Factor alpha (TNF-alpha), and Nitric Oxide Synthase 2 (NOS2) has been reported to correlate with favorable clinical outcomes in many human cancers [21,22,23]. However, this binary M1/M2 classification is too simplistic to explain the phenotype and functions of macrophages in tumors because macrophages in tissue are highly heterogeneous with dynamic and extremely plastic phenotypes and functions, which are continuously shaped by their tissue microenvironment.

In the GBM, TAMs have a distinct protumor role and their accumulation correlates with tumor grade [24,25,26,27]. Functionally, TAMs produce low levels of inflammatory cytokines and lack the ability to induce T-cell responses [28]. The number of tumor-associated macrophages (TAMs) inversely correlates with overall survival in patients with recurrent glioblastoma [27,29]. Studies in mice showed that TAMs mediate tumor recurrence in mice [30,31,32]. A recent study showed that radiation induced acquisition of a recurrence-specific phenotype in TAMs, which supports glioma proliferation and regrowth [33]. The targeting of TAMs using CSF-1R inhibition blocked the acquisition of this pro-tumorigenic phenotype and enhanced initial glioma-debulking effects of radiotherapy.

**Figure 1:**
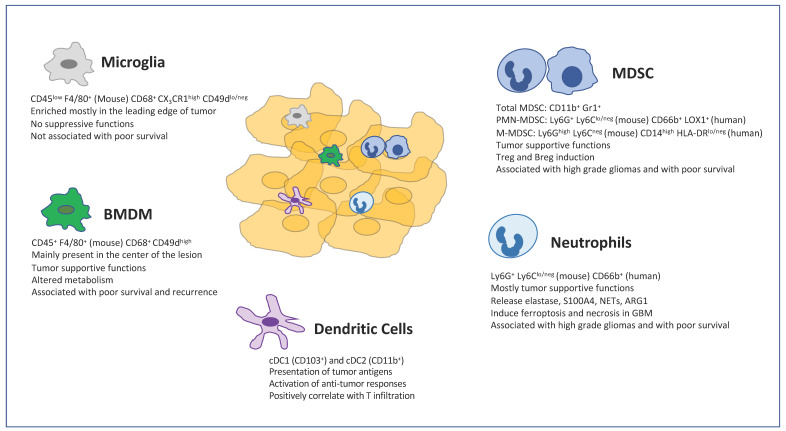
Myeloid cell compartment in the GBM tumor microenvironment. Surface molecules for the identification of myeloid cells are shown along with main characteristics and functions.

TAMs largely originate from bone marrow-derived monocytes [34,35]. In brain tumors, TAMs consist of a mixture of bone marrow-derived macrophages (BMDMs) and resident macrophages known as microglia (MG). Whereas BMDMs are mostly differentiated from tumor infiltrating monocytes, MG are derived from erythro-myeloid progenitors (EMPs) developed in the yolk sac, which migrated into the CNS at the embryonic stage [36] (Figure 1).

## 3. Distinctive Features of MG and BMDMs

Despite MG and BMDMs being two ontogenetically distinct myeloid cell populations, they share immune regulatory features and the expression of common markers, such as CD11b, CD68, ionized calcium-binding adapter molecule 1 (IBA1), and F4/80 (mouse specific) [37,38,39]. Commonly, microglia cells are described as CD11b+/CD45low (or CD45int), whereas the BMDMs are defined as CD11b+/CD45 high population [40]. However, the definition of microglia and BMDMs by their CD45 expression level is inadequate since activated microglia can rapidly upregulate CD45 expression [41]. Genome-wide microarray and single-cell RNA sequencing analyses have allowed for phenotypic and transcriptomic differentiation between these two populations. Microglia are characterized by low expression of major histocompatibility complex II (MHCII), the absence of C-C motif chemokine receptor 2 (CCR2), and the high expression of purinergic receptor P2RY12 and C-X3-C motif chemokine receptor 1 (CX3CR1), while blood-derived macrophages demonstrate high expression of CD45, MHCII, and tyrosine-protein kinase Mer (MERTK). Recently, the transmembrane protein 119 (TMEM119) and the glycoprotein Itga4 (CD49D) were reported as novel microglia-reliable markers to discriminate resident microglia from BMDMs in both humans and mice [42,43].

Single cell RNA sequencing (scRNA-seq) studies underlined that BMDMs in gliomas have a distinct phenotype and regional distribution compared to MG. Pinton et al. showed that BMDMs exhibit characteristics of pro-tumoral cells exerting a strong immunosuppression, while MG showed little to no suppression. More interestingly, they highlight that beyond functional differences, BMDMs were unique in oxidative and iron metabolism and phagocytosis [44]. Contrary to MG, the high frequency of BMDMs in gliomas and the elevated expression of BMDMs-related genes significantly correlated with the clinical outcome of glioma patients and with significantly poor overall survival in grade II–III low grade gliomas, respectively [13,45]. Poon et al. found out that there are significantly fewer TAMs in untreated human IDH-mutant GBMs than the wild-type (WT) and they are more pro-inflammatory, suggesting their contribution to the better prognosis of these tumors [46]. In IDH-mutant patients, a decrease in median overall survival was associated with enrichment of the BMDMs IDH WT signature, whereas IDH WT patients with a low enrichment score showed increased survival [12]. The frequencies of BMDMs observed in the IDH1 WT glioma tumor microenvironment (TME) was higher than BMDMs frequency in the TME of IDH1-mutant gliomas, which is associated with better prognosis. CNS-invading phagocytes in the TME of IDH1-mutant glioma were predominantly composed of monocytes and low frequencies of BMDMs [13]. The accumulation of BMDMs in the TME of IDH1 WT glioma was O6-independent of the methylation status of the O6-methylguanine DNA methyltransferase (MGMT) promoter [13].

## 4. Localization of MG and BMDMs

The recruitment of MG and BMDMs in the surroundings of the tumor is controlled via the release of several chemo-attractants, including fractalkine (CX3CL1) whose receptor, CX3CR1, is mostly expressed by microglia in adults [47].

Circulating monocytes are recruited by several tumor-derived chemo-attractants as CCL2 (MCP-1), CCL3 (MIP-1), CXCL12 (SDF-1), and CSF-1 at tumor sites where they differentiate into TAMs and facilitate tumor progression [48,49,50]. Chemoattraction by osteopontin (OPN) was also recently reported in GBM, binding to macrophage-expressed integrin αvβ5 [51].

The infiltration of BMDMs increases from the periphery to the center of the glioma lesion and is almost absent in the marginal area [44]. In this respect, Muller et al. found out that BMDMs are enriched in perivascular and necrotic regions of tumor, while MG are enriched in the leading edge of tumor infiltration [45]. In particular, MG (Iba1^+^ CD163^−^) were diffusely scattered throughout gliomas, while Iba1^+^CD163^+^ BMDMs and CD206^+^, CD169^+^, and CD209^+^ subsets of BMDMs were found in close proximity to blood vessels in gliomas [13].

Klemm et al. also showed a significant enrichment of MG and BMDMs in the perivascular niche [12]. However, compared with MG, BMDMs were distributed to a much closer proximity to CD31+ vascular structures. Recently, it has been shown that CD163+ cells were the most common cell type in both the peritumoral area (PTA) and tumor core (TC) in GBM patients. Indoleamine-pyrrole 2,3-dioxygenase (IDO) and programmed death-ligand 1 (PDL-1) were predominantly expressed in TC, probably indicating a more suppressive environment in close proximity of the TC [52].

## 5. Epigenetic and Transcriptional Features of MG and BMDMs

The transcriptomic and epigenetic profile of TAMs can be shaped by numerous factors present in the GBM microenvironment. The identification of specific markers for the separation of distinct populations of TAMs allowed for a more precise characterization of the molecular features of BMDMs and MG.

The analysis of transcriptome of TAM from preclinical models of GBM revealed distinctive transcriptional profiles of MG and BMDMs that do not correspond to the traditional M1/M2 classification scheme [53]. The authors demonstrated that the expression of Gpnmb and Spp1 (osteopontin) is highly upregulated in both murine and human glioma-associated microglia/macrophages. Gpnmb and Spp1 are implicated in immunosuppression and tumor cell invasion, respectively. High expression of these genes has been associated with poor prognosis in human GBM.

A transcriptomic analysis showed differences between BMDMs versus MG and identified several differentially expressed genes enriched in BMDMs compared to MG, including genes related to effector functions, suppressive molecules, chemokine involved in wound healing, and antigen presentation and costimulation. Besides a distinctive tumor-specific profile, BMDMs and MG show differences in genes that underline their differential ontogenesis [43]. Moreover, MG and BMDMs unique transcriptomic profiles and shared expression signatures are further influenced by the type of the disease, such as IDH mutant versus IDH WT glioma, and glioma versus brain metastasis (BrMs) [13].

The epigenetic states lead to differential TAMs education between MG and BMDMs and influence their stimulus-dependent transcriptional induction [43]. Indeed, binding sites for PU.1, a critical transcriptional factor for the development and functions of myeloid cells, are already altered at enhancers and promoters for the genes specific to MG and BMDMs. Therefore, BMDMs and MG might be poised to engage in distinct transcriptional networks based on initial enhancer selection. Since differential expression of binding partners influences PU.1 genomic occupation, binding partners that are absent in MG and expressed in BMDMs can sculpt genomic PU.1 occupancy and play a role in brain tumors [43]. The study of the dynamic regulatory networks of blood-derived TAMs in GBM has indeed recently identified a macrophage receptor with collagenous structure (MARCO) that promotes proliferative activities and therapeutic resistance to irradiation of glioma stem cells as well as tumor engraftment and growth in vivo [54]. Moreover, therapeutic strategies such as radiotherapy may alter the transcriptional characteristics of TAM subpopulations over time. Akkari et al. found that radiotherapy elicits an initial transient antitumor response associated with a progressive accumulation of TAMs in gliomas, where relative proportions of MG and BMDMs are altered and gene expression signature converge towards a common suppressive phenotype [33].

Currently, a report supports the idea that the contribution of epigenetic mechanisms to glioma-induced “transcriptional memory” in TAMs results in a tumor-supportive phenotype. Cultured microglia pre-exposed to glioma-conditioned medium (GCM) acquire a “transcriptional memory”, displaying reduced expression of inflammatory genes after re-stimulation with lipopolysaccharide. GCM induces the expression and enzymatic activity of histone deacetylases (HDAC), leading to erasure of histone acetylation and the acquisition of repressive histone marks (H3K27 trimethylation) at inflammatory genes, which correlates with silencing of their expression. HDAC inhibitors block GCM-induced histone modifications, restoring microglia ability to initiate effective inflammatory responses [55].

## 6. Metabolic Profile of MG and BMDMs

The tumor microenvironment (TME) is characterized by high nutrient competition, low pH, limited oxygen, and high accumulation of metabolites. Such hostile conditions result in metabolic changes that are needed to fulfill the energy requirements of tumor and immune cells. Myeloid cells, including suppressive and pro-tumoral cells, show a high metabolic plasticity, which is a driver of their differentiation and function in cancer. During recent years, the targeting of metabolic pathways represents a promising approach to reprogram functions of myeloid cells and thus to improve the TME for cancer immunotherapy [56].

Macrophages adjust their phenotype and function in response to local cues provided by the TME [57]. Macrophages rely on different metabolic pathways such as glycolysis, glutaminolysis, or fatty acid oxidation (FAO) to maintain critical cellular functions [58,59]. Glucose and its oxidative metabolism are responsible for most of the cellular energy produced in the brain; however, lactate, pyruvate, ketone bodies, and glutamate may be used under certain circumstances [60,61,62].

The microenvironment of the tissue drives significant changes in the preferred metabolic pathways that regulate the polarization of macrophages: proinflammatory states increase reliance on glycolysis and glutaminolysis, whereas anti-inflammatory phenotypes increase OXPHOS and possibly FAO. In gliomas, a decreased glycolytic metabolism in BMDMs from human gliomas compared to MG, is associated with increased immunosuppression in the TME and poor patient survival [45]. Oncometabolites in the tumor microenvironment act on TAMs and other immune cells to facilitate tumor growth. For example, lactate produced by tumor cells induces pro-tumorigenic TAMs through a mechanism mediated by HIF1α [63]. Kynurenine produced by glioblastoma cells activates aryl hydrocarbon receptor (AHR) in TAMs to modulate their function and T cell immunity. AHR promotes CCR2 expression, driving TAM recruitment in response to CCL2. AHR drives the expression of the ectonucleotidase CD39 in TAMs, which promotes CD8+ T cell dysfunction by producing adenosine in cooperation with CD73 [64].

On the other hand, TAMs reinforce the metabolic shift of GBM cells to aerobic glycolysis through IL-6 which enhances the activity of phosphoglycerate kinase 1 (PGK1) by promoting its phosphorylation [65].

In summary, although MG and BMDMs share the same microenvironment, they have distinct metabolic profiles. These metabolic differences may drive unique transcriptional and epigenetic changes that may inform the divergent functions of various populations of TAMs in GBM.

## 7. Therapeutic Targeting of TAMs

Most therapeutic approaches targeting glioma cells have failed [7,66]. An alternative strategy is to target the more genetically stable stroma in the glioma microenvironment. Because of their abundance, TAMs represent an ideal target for therapy.

Standard of care treatment includes surgery, radiotherapy, and temozolomide (TMZ) chemotherapy. As immunotherapeutic options for GBM patients are being expanded and investigated, the role of temozolomide and radiotherapy as a combinatorial strategy with immunotherapy will become increasingly relevant. Temozolomide, traditionally used for direct antitumor effects, has immunomodulatory properties [67]. Lymphoablative doses of TMZ were shown to increase tumor antigen-specific immune responses in GBM patients [68,69] and GBM-bearing mice [70]. However, in the context of TAM, TMZ can be detrimental. Indeed, TMZ alone or in combination with radiotherapy induced a more pro-tumorigenic phenotype in macrophages [71,72]. Reports indicate that radiotherapy alone favored myeloid cell recruitment [73,74], and rapidly induced a heterogeneous pro-tumorigenic phenotype in BMDM and MG [33] in a pre-clinical model of GBM. Since chemotherapy and radiotherapy have both beneficial and detrimental effects on phenotype and functions of different immune cells, these effects have to be considered when designing combined therapies.

**Figure 2:**
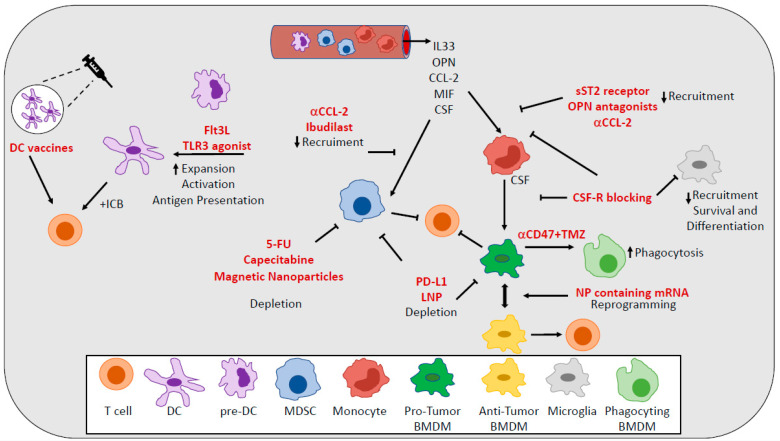
Targeting myeloid cells in GBM. Different approaches aimed to deplete and to inhibit functions and recruitment of MDSCs and TAMs are used to reduce the immunosuppression. DC vaccines and approaches aimed to increase accumulation, activation, and functions of DCs are used to stimulate effector functions.

A variety of approaches have been explored as means to either reduce TAMs numbers or to reprogram them to be more inflammatory and immunogenic [75] (Figure 2). Since colony stimulating factor-1 (CSF-1) secreted by glioma cells [76] is a driver of differentiation, polarization, survival, and recruitment of TAMs, strategies aimed at targeting the CSF-1 receptor (CSF-1R) on TAMs have been extensively investigated [77,78]. A specific inhibitor of CSF-1R significantly altered macrophage polarization in a mouse model of GBM, suppressing the expression of M2 markers without depleting TAMs [79]. CSF-1R inhibition has been shown to increase survival by blocking proneural glioma progression and resulting in the regression of established tumors [79]. However, Quail et al. found that although CSF-1R blockade prolongs survival in mouse models of GBM, more than 50% of tumors eventually recurred. Recurrence is correlated with elevated PI3-K activity in tumors, driven by macrophage-secreted IGF-1. Blocking PI3-K and IGF-1 signaling in rebounding tumors prolongs survival [11]. Targeting TAMs populations using a CSF-1R inhibitor combined with radiotherapy substantially enhanced survival in preclinical models, changing the relative abundance and phenotypes of MG and BMDMs [33]. CSF-1R inhibition can block the radiotherapy-induced alternative activation in MG and BMDMs and thus the acquisition of recurrence-specific phenotypes in these cells, which support glioma proliferation and regrowth [33]. Despite the promising results in pre-clinical models, no objective response was observed in a phase II study (NCT01349036) of a CSF1R inhibitor in patients with recurrent glioblastoma [7,80].

More recently, another study showed how the secretion of IL-33 from glioma cells and the presence of nuclear IL-33 within them initially act to recruit monocytic cells with an M1-like antitumorigenic phenotype from circulation and then, favor the reprogramming of the TAMs to a pro-tumorigenic M2-like phenotype that in turn fuels rapid glioma growth [81]. Therapeutic strategies using soluble ST2 receptors to sequester secreted IL-33 [82] might provide a benefit by maintaining a TME with TAMs of a tumor-suppressive phenotype.

A genetic reprogramming of macrophages to perform antitumor functions without causing systemic toxicity might be achieved using targeted nanocarriers that can deliver in vitro-transcribed mRNA encoding M1-polarizing transcription factors through the mannose receptor CD206. Infusions of nanoparticles containing mRNAs encoding interferon regulatory factor 5 in combination with its activating kinase IKKβ not only inhibit the immunosuppressive state of TAMs but also reprogram them to an antitumor phenotype inducing immunity and tumor regression. These nano reagents are safe for repeated dosing and might be used in the clinic to avoid systemic treatments that disrupt immune homeostasis [83].

OPN represents another important chemokine for recruiting macrophages to glioblastoma and for maintaining the M2 macrophage gene signature and phenotype. OPN-deficient mice intracerebrally implanted with GL261 have significantly prolonged survival relative to WT mice. This OPN deficiency is associated with reduced immune-suppressive M2 macrophages infiltration, especially within the local TME, and markedly enhanced immune antitumor effector function in both CD4+ and CD8+ T cells in spleen, blood, and brain tumors. From a therapeutic perspective, OPN inhibitors/antagonists (such as OPN-specific antibodies and aptamers) could be considered as potential agents for treating cancer and other types of diseases in which there is overactive OPN signaling [51].

Glioma cells can evade phagocytosis by upregulating anti-phagocytosis molecule CD47; however, CD47 blockade alone is inefficient in stimulating glioma cell phagocytosis by macrophages. The combination of CD47 inhibition with temozolomide (TMZ) results in a significant pro-phagocytosis effect enhancing antigen cross-presentation and resulting in more efficient T cell priming. This therapeutic combination inhibits glioma growth, but also activates an immune checkpoint that can be turned off by sequential administration of an anti-PD1 antibody [84].

Immunotherapy and especially immune checkpoint inhibitors and programmed cell death (PD)-1/PD-L1 inhibitors have transformed the landscape of cancer treatment and improved patient survival in a number of different cancer types. However, no clinical benefit has been observed in GBM patients, mainly due to several mechanisms of resistance to therapy present in GBM TME [85]. Goswami et al. identified a unique population of CD73^hi^ TAMs in GBM that persists after anti-PD-1 treatment, as a potential mechanism of resistance. The absence of CD73 improved survival in a murine model of GBM treated with anti-CTLA-4 and anti-PD-1. CD73 represents a new specific immunotherapeutic target to improve antitumor immune responses to immune checkpoint therapy in GBM [86]. A lipid nanoparticle (LNP) formulation, surface-functionalized with an anti-PD-L1 therapeutic antibody (αPD-L1), was capable of actively targeting and delivering dinaciclib, a cyclin-dependent kinase inhibitor, to mouse and human tumor-associated myeloid cells (TAMCs) by recognizing highly expressed PD-L1 in myeloid cells, including BMDMs, microglia, and myeloid-derived suppressor cells. PD-L1-targeted LNPs led to a robust depletion of TAMCs and an attenuation of their immunosuppressive functions. The delivery efficiency of PD-L1-targeted LNPs was robustly enhanced in the context of radiation therapy (RT) owing to the RT-induced up-regulation of PD-L1 on glioma-infiltrating TAMCs [87].

Based on recent evidence, it is clear that TAMs can be successfully targeted. Unfortunately, to date, the clinical application of these approaches has been modest. This limited success may be due to the fact these therapeutic strategies were generally focused on targeting the total TAMs pool. It is evident that TAMs are plastic as well as metabolically and functionally heterogenous. Further the understanding of the mechanisms of such dynamic heterogeneity displayed during tumor progression and responses to therapy will help to define more efficient strategies for their therapeutic targeting in GBM.

## 8. Myeloid Derived Suppressor Cells (MDSCs) in GBM

MDSCs are a heterogeneous population of cells generated during a large array of pathologic conditions ranging from cancer to obesity. MDSCs are a critical component of the suppressive network that supports tumor progression and contribute to the resistance of therapy. MDSCs consist of two large groups of cells: granulocytic or polymorphonuclear MDSCs (PMN-MDSCs) and monocytic MDSCs (M-MDSCs). PMN-MDSCs are phenotypically and morphologically similar to neutrophils, whereas M-MDSCs are similar to monocytes [88]. However, PMN-MDSCs and M-MDSCs are characterized by a distinct set of genomic and biochemical features, and by the ability to suppress immune responses (Figure 1).

Numerous mechanisms by which MDSCs inhibit immune responses have been reported, including inhibition of the antitumor activity of cytotoxic T cells [89], suppression of NK cell [90], macrophage and dendritic cell function [91], and induction of Tregs and Bregs [92,93]. ARG1, nitric oxide (NO), and PGE2 [94] are major contributors to MDSCs’ immune suppressive activity [88]. M-MDSCs may also contribute to the pool of TAMs in GBM and predominantly to BMDMs. It is known that after migration to a tumor site, M-MDSC rapidly differentiate to TAMs [95,96], and monocytes or M-MDSC are essential for TAM accumulation [97]. In the tumor microenvironment, HIF-1α facilitated the differentiation of M-MDSCs into TAMs, through a mechanism involving CD45 tyrosine phosphatase activity and down-regulation of STAT3 activity [96,98].

The important role of MDSCs in cancer is now widely recognized and their presence correlates with a negative clinical outcome in patients [88,99]. In glioma patients, the intratumoral and systemic blood frequency of MDSCs increases during glioma progression and correlates with the grade of glioma malignancy and prognosis [100,101,102,103]. GBM patients with extended survival also had reduced MDSCs, similar to the levels of low-grade glioma (LGG) patients [104]. The increased MDSC accumulation at the time of recurrence predicts poor GBM outcome in patients [104]. However, the role of MDSC in GBM recurrence is not well understood and further studies are required to clarify this critical point. In mouse models of GBM, the proportion of M-MDSC is higher than the proportion of PMN-MDSC [105,106]. In human gliomas, it is unclear whether there is the predominance of a specific subset of MDSCs. Several studies reported that both subsets of MDSC can be identified in the blood and tumors of GBM patients [100,101,102,103]. Indeed, a study showed that the majority of the MDSCs were PMN-MDSCs in blood of GBM patients [100]. Gielen et al. found that MDSCs were significantly increased among peripheral blood mononuclear cells from patients with GBM and MDSCs found in tumor tissue were almost exclusively PMN-MDSCs [102]. Dubinski et al. also revealed that the frequency of M-MDSCs and PMN-MDSCs was significantly higher in the peripheral blood of GBM patients compared with healthy donors [101]. Furthermore, Raychaudhuri et al. found that the proportion of PMN-MDSC was higher than M-MDSC subtypes in human GBM tumors [106]. A recent study pointed out that the accumulation of specific subsets of MDSCs in a mouse model and patients with GBM can be driven by sex dimorphism. Whereas M-MDSCs were enriched in the male tumors, PMN-MDSCs were elevated in the blood of females. A high PMN-MDSC/IL1β gene signature correlated with poor prognosis in female patients [107]. In summary, all of the above studies indicate that different MDSC subsets can accumulate in the blood and tissue of GBM patients. These data suggest that different subsets of MDSCs may have different roles in GBM. The recent discovery of new markers, such as LOX1 [108], CD84 [109], and CXCR1 [110] for the identification of MDSCs and for the separation of MDSCs from normal monocytes and neutrophils, will help to further characterize these populations, clarify their role and the mechanisms underlying their accumulation in GBM patients.

## 9. Therapeutic Targeting of MDSCs

MDSCs represent a critical component of immunosuppression that supports tumor progression and resistance to therapy and several different therapeutics have been investigated, aimed at manipulating accumulation, differentiation, functions, and migration of MDSCs in brain cancers. Since many different strategies to target MDSCs have been intensively described in several previously published reviews [88,111], we will discuss the therapeutic targeting of MDSCs in GBM.

Several attempts to deplete MDSCs or to inhibit recruitment and functions of MDSCs have been described in pre-clinical models and in patients with GBM (Figure 2). The depletion of circulating MDSCs using 5-flurouracil (5-FU) resulted in increased frequencies of activated T cells and prolonged survival in pre-clinical models [112]. In patients undergoing surgery for recurrent GBM, the depletion of MDSCs using metronomic capecitabine increased cytotoxic immune infiltration in brain tumors [113]. Another study showed that TMZ chemotherapy may also be used to deplete MDSC. TMZ selectively killed monocytes but not macrophages and dendritic cells in vitro [114]. Since BMDM differentiate from monocytes and M-MDSC are relatively immature monocytes with suppressive functions, TMZ may be used to selectively deplete M-MDSC and reduce the proportion of BMDM in the TME. However, studies in vivo are required to test this hypothesis. Radiotherapy (RT) can also regulate the accumulation of MDSC in cancer. For instance, high-dose ablative hypo-fractioned RT (ABHRT) rather than multiple lower dose fractionated radiotherapy (CFRT) reduced the proportion of MDSC in tumor-bearing mice [115,116,117].

The specific depletion of PMN-MDSCs extended survival in female mice but no benefit was observed in male mice, indicating that the female patients could benefit from the elimination of PMN-MDSCs [107].

The inhibition of MDSCs recruitment at the tumor bed showed promising results in mouse models. In syngeneic and intracranial xenograft mouse models with GL261 glioma, administration of an anti-CCL2 antibody could block recruitment and decrease the number of both MDSCs and TAMs in the TME, leading to prolonged survival of tumor-bearing mice [118,119]. Moreover, CCL2 blockade in combination with the current standard TMZ-based chemotherapy also prolonged the survival of mice with glioma [118].

The chemo-attractant macrophage migration inhibitory factor (MIF) produced by glioma stem cells also favor the recruitment of MDSCs, as well their functions through the regulation of ARG1 expression. The targeting of MIF indeed conferred a survival advantage to tumor-bearing mice and increased the cytotoxic T cells within the tumors. A recent study reported that M-MDSCs expressed high levels of the MIF cognate receptor CD74 and the targeting of M-MDSCs with ibudilast, a brain penetrant MIF-CD74 interaction inhibitor, reduced MDSCs function and enhanced CD8 T cell activity in the TME [120].

The treatment of mice with magnetic nanoparticle-based platform with cationic polymer modification to promote radiotherapy for glioma treatment induced cytotoxicity to glioma cells under radiation as well as significant survival benefits in both immunocompetent and athymic mice with glioma. The efficacy was attributed to the destruction of glioma cells as well as MDSCs’ repolarization from an immunosuppressive phenotype to a pro-inflammatory phenotype [121].

Since differences in gender drive differences in the accumulation and functions of MDSCs, Bayik et al. recently proposed that M-MDSCs could be targeted with antiproliferative agents in males, whereas PMN-MDSCs function could be inhibited by IL1β blockade in females [107].

In summary, the presence of MDSCs is associated with poor prognosis in GBM patients and the therapeutic approaches have shown promising results in the context of GBM, indicating that the targeting of MDSCs may be feasible and effective.

## 10. Neutrophils in GBM

Neutrophils are the most abundant circulating leukocytes in humans and they have emerged for their wider functions in the immune response [122]. Neutrophils are growingly appreciated as a critical component of the TME. Neutrophils systemically and intratumorally accumulate in glioma patients and a high frequency of neutrophils is negatively associated with the prognosis of GBM patients [123,124,125,126] (Figure 1). Moreover, the number of tumor infiltrating neutrophils correlates with glioma grade and represents a negative prognostic parameter for resistant patients [127,128].

Neutrophils can be commonly found in the tumor core of GBM [7]. IL8, MIF, and CXCL8 lead to neutrophils infiltration at the tumor site [129,130]. Once in the TME, neutrophils release elastase that facilitate neutrophils and glioma cancer cell infiltration at the tumor site [131]. Neutrophils directly promoted GBM-initiating cells’ proliferation and migration via the production of S100A4, which induced the transition to a mesenchymal phenotype, favoring cancer invasion and resistance to anti-VEGF therapies [132]. Furthermore, neutrophils form neutrophil extracellular traps (NETs) that protect cancer cells in the brain and support the development of both primary tumors and metastasis [133]. Moreover, neutrophils from GBM patients suppressed T-cell proliferation in an ARG1 dependent manner [134]. Recent evidence showed that tumor damages occurring during early tumor progression may also favor the recruitment of neutrophils to the tumor site and that neutrophils-induced ferroptosis promoted tumor necrosis in glioblastoma progression through a mechanism involving iron-dependent accumulation of lipid peroxides within the tumor [108,135]. Analyses of human GBMs supported that neutrophils and ferroptosis are associated with necrosis and predict poor survival.

## 11. Neutrophils and PMN-MDSCs: Two Sides of the Same Coin

It is well accepted that different subsets of neutrophils can coexist in the same cancer patient [88,108,136]. Such heterogeneity in the context of cancer is dependent on cancer types, tumor progression, and neutrophils maturation stage [137,138,139,140]. At any given moment, myeloid cells in tissues comprise classically activated neutrophils with pro-inflammatory and antitumor activity and pathologically activated MDSCs with potent immune suppressive and pro-tumorigenic activity. Despite the fact that PMN-MDSCs and neutrophils are different at biochemical, genomic, and functional levels, polymorphonuclear neutrophils (PMNs) were all classified as either neutrophils or PMN-MDSCs in most of the studies on glioma patients. This limits the clinical relevance of these cells in GBM. However, a recent study, using the LOX1 marker, which has emerged as a specific marker of human PMN-MDSCs [108], started to shed light on the role of neutrophils and PMN-MDSCs in GBM patients. Chai et al. reported that the high presence of LOX1+PMNs but not LOX1-PMNs in the peripheral blood and tissue of GBM patients negatively correlated with the numbers of effector immune cells in GBM patients and was associated with an early recurrence and disease progression [141]. LOX1+PMNs displayed a PMN-MDSCs profile, with a significant increase in the expression of reactive oxygen species (ROS), ARG1, and iNOS, and the ability to suppress the proliferation of CD3+ T cell in an ARG1/iNOS-dependent manner [141]. In summary, as shown already in other tumors, neutrophils and PMN-MDSCs can coexist in GBM and thus may have different functions in brain tumors. The understanding of the phenotypic and functional heterogeneity of neutrophils will further clarify their contribution to immune and therapeutic responses to GBM, as well as their clinical relevance.

## 12. Dendritic Cells in GBM

Dendritic cells (DCs) are a diverse group of specialized antigen-presenting cells (APC) with a key role in the initiation and regulation of innate and adaptive immune response [142] (Figure 1). DCs differentiate through subsequent steps inside bone marrow starting from common myeloid progenitors (CMP) and then macrophage/DC progenitors (MDP). From MDPs, both common DC precursors (CDP) and common monocytes precursors (cMOP) can be derived [143,144]. Conventional DCs derive from CDP and comprise two main subsets: CD8α^+^ and/or CD103^+^cDC1s and CD11b^+^cDC2s. Plasmacytoid DC (pDCs) developed from both CDP and lymphoid progenitors, giving rise to two different pDCs subsets. During inflammatory conditions, monocytes can give rise to monocyte-derived DCs (MoDCs) in the tissue [145].

In homeostatic conditions, DCs are not present inside the brain parenchyma. However, during pathological conditions such as neurodegenerative diseases and cancer, DCs can reach the brain tissue via afferent lymphatic vessels or endothelial venules [146].

The cDC1s have a pivotal role in antitumor immunity and in the success of immunotherapy [91,147,148,149,150,151]. However, the specific role of cDCs in the setting of GBM has yet to be elucidated. In brain tumors, it is suggested that DCs recognize and present tumor-derived antigens inside the brain tissue or in the draining lymphoid stations in order to elicit a T effector cell response against cancer cells [152,153]. Two recent papers showed that DCs with a signature of cDC1s and with an increased phagocytic ability were present within the brain tumor TME [12,13]. In glioma, an increase in the relative frequencies of T cells, neutrophils, and pDCs correlated negatively with TAM/monocyte frequencies, whereas T cell frequencies positively correlated with pDCs and cDCs frequencies [13].

It is well accepted that tumors affect infiltration, differentiation, recruitment, survival, and functions of DCs via diverse mechanisms [154]. Tumor-derived factors and metabolites produced by other immune cells also affected the functionality of cDCs in cancer. For example, the accumulation of MDSC-derived oxidized lipids in cDC1s was implicated in the negative regulation of their ability to cross-present tumor antigens [91,147]. In GBM, fibrinogen-like Protein 2 (FGL2), produced by tumor cells, interfered with GM-CSF signaling, blunting the differentiation of CD103+ cDC1s and consequently, lowering the CD8+ T-cell response [155]. PGE2 from glioma cells was found to increase the expression of IL-10 by DCs, in turn leading to the induction of a regulatory response in CD4 T cells and a reduced stimulation of effector lymphocytes [99]. Recent explorations into the role of DCs in glioma progression have focused on homeostatic regulators of DC function including Nrf, a redox-sensitive transcription factor that is involved in counteracting the effects of reactive oxygen species. The TME of GBM induces overexpression of Nrf in DCs, which in turn results in the suppression of DCs maturation and the consequent decrease in effector T cell activation. The inhibition of Nrf2 pathways rescues maturation of CD80+ and CD86+ DCs in glioma-cell-conditioned medium and partially restores secretion of bioactive cytokines such as IL-12p70 [156].

## 13. Therapeutic Targeting of DCs

The clinical success of DC therapies, including DC-based vaccines, in other cancers has led to increasing interest in the use of dendritic cells to fight brain tumors [154] (Figure 2). Despite the encouraging results coming from pre-clinical and Phase I studies, these DC-based vaccines still struggle in showing a clinical benefit in GBM patients [157]. Recent advances in DC vaccines include preconditioning of the vaccine site. In a high-profile study, DCs loaded with Cytomegalovirus (CMV) phosphoprotein 65 (pp65) RNA experienced significant improvements to lymph node homing and prolonged patient overall survival after the vaccine site was preconditioned with tetanus/diphtheria [158]. The results from three separate clinical trials utilizing CMV-specific DC vaccines showed exceptional long-term survivors in patients with newly diagnosed GBM [159].

Several factors may limit the efficacy of current DC vaccines, including tumor antigen loading, the ability of DCs to migrate, and the remarkable local and systemic immunosuppression in GBM. DC vaccines may be more successful in combination with other immunotherapies. DC vaccination may be used to counteract the lack of efficacy seen with immune checkpoint blockade in tumors, such as GBM, with low mutational burdens. Moreover, therapeutic agents aimed at lowering the immunosuppression in GBM TME would be beneficial for the effectiveness of DC-based vaccines.

Given the labor- and time-intensive process of generating DC vaccines ex vivo in addition to variable response rates, efforts are underway to develop therapies that target DCs in vivo. Targeting DCs in vivo may circumvent the issue seen with ex vivo-generated DCs [160,161] and allow for the specific targeting of specialized subsets of DCs in the TME. The expansion and activation of DCs in vivo also represent alternative promising approaches to target DCs in cancer. In a preclinical model of GBM, the activation of DCs using TLR3 agonists indeed enhanced the antitumor immune response to checkpoint blockade and increased the survival of mice. The survival benefit achieved by combination of anti-PD-1 therapy with Poly(I:C) [162] was partially reduced following DC depletion. The administration of Flt3L increased the numbers of tumor-infiltrating CD103+cDC1s and enhanced the efficacy of checkpoint blockade in GBM models [163]. The feasibility of this approach for GBM patients may come from the results of a study in indolent non-Hodgkin’s lymphomas, which are incurable with standard therapy and are poorly responsive to checkpoint blockade. According to this study, the combination of Flt3L, radiotherapy, and a TLR3 agonist induced the recruitment of antigen-loaded and activated intra-tumoral, cross-presenting DCs. This combination induced antitumor CD8+ T cell responses and enhanced the PD1-blockade efficacy [164], in a cross-presenting DCs-dependent manner.

Another strategy to increase the numbers of DCs comes from the injection of hematopoietic stem and progenitor cells (HSC) that are shown to generate intratumoral DCs that potentiate T-cell responses and promote glioma rejection [165].

## 14. Conclusions and Perspective

Growing evidence has highlighted the pivotal role of myeloid cells in the brain tumor microenvironment. It is becoming clear that, as shown in different cancer types, the composition of myeloid cells in the TME critically contributes to the success of immunotherapy as well as adjuvant treatments such as radiation and chemotherapy. The GBM immune microenvironment is populated by myeloid cells, including TAMs, MDSC, neutrophils, and DCs. Suppressive and pro-tumorigenic myeloid cells that represent a vast majority of myeloid cells in GBM TME actively contribute to the resistance of GBM to immunotherapy. Unfortunately, to date, the clinical application of therapeutic manipulation of myeloid cell compartment and immunosuppression, which have shown promising results in preclinical models, has been very modest. The recent advances in the understanding of mechanisms of myeloid cell-driven immunosuppression as well as of mechanisms of recruitment and localization of myeloid cells will be beneficial when designing new therapeutic approaches. However, there still are largely unanswered questions regarding how GBM governs metabolic and epigenetic landscapes of myeloid cells, as well as the mechanisms of the dynamic heterogeneity of these cells during immune and therapeutic responses in the context of GBM. Moreover, the recognized presence of DCs, known for their pivotal role in antitumor immunity and in the success of immunotherapy, in the GBM TME, demands for a comprehensive examination of their functions and roles, as well as potential mechanisms of their dysfunction.

Radiotherapy and chemotherapy can have both beneficial and detrimental effects on phenotype and functions of the immune system. Changes in phenotype and functions of immune cells could certainly affect the outcome of immunotherapy following radio/chemotherapy and they should be considered when designing any combination therapy. Moreover, dosage of chemotherapy and timing of immunotherapy following chemotherapy have to be further investigated to lead to the design of novel therapeutic strategies and improve synergy in combined treatments.

In the next few years, we will witness more substantial advances in the understanding of the complex biology of myeloid cells and of their interplay with other cell components of the GBM TME. We highly believe that such new knowledge is the prerequisite to produce more effective cures for GBM patients.
